# Status epilepticus in adults: From etiology to treatment response

**DOI:** 10.1002/epi.70446

**Published:** 2026-08-13

**Authors:** Simona Lattanzi, Giada Giovannini, Niccolò Orlandi, Francesco Brigo, Eugen Trinka, Stefano Meletti

**Affiliations:** ^1^ Neurological Clinic, Department of Experimental and Clinical Medicine Marche Polytechnic University Ancona Italy; ^2^ Neurophysiology Unit and Epilepsy Center Ospedale Civile di Baggiovara, Azienda Ospedaliero‐Universitaria Modena Modena Italy; ^3^ Department of Biomedical, Metabolic, and Neural Science, Center for Neuroscience and Neurotechnology University of Modena and Reggio Emilia Modena Italy; ^4^ Innovation, Research, and Teaching Service Teaching Hospital of the Paracelsus Medical University, Azienda Sanitaria Dell'alto Adige Bolzano‐Bozen Italy; ^5^ Department of Neurology, Neurocritical Care, and Neurorehabilitation, Christian Doppler University Hospital, Center of Cognitive Neuroscience Paracelsus Medical University, member of the European Reference Network EpiCARE Salzburg Austria; ^6^ Neuroscience Institute, Christian Doppler University Hospital, Center of Cognitive Neuroscience Paracelsus Medical University Salzburg Austria; ^7^ Karl Landsteiner Institute for Clinical Neuroscience Salzburg Austria; ^8^ Institute of Public Health, Medical Decision‐Making, and Health Technology Assessment UMIT–Private University for Health Sciences, Medical Informatics, and Technology Hall in Tyrol Austria; ^9^ Neurology Department Ospedale Maggiore, Azienda Ospedaliero‐Universitaria Parma Parma Italy; ^10^ Department of Medicine and Surgery University of Parma Parma Italy

**Keywords:** acute symptomatic, antiseizure medications, refractory, responsive, superrefractory

## Abstract

This study explored the association between etiology of status epilepticus (SE) and treatment responsiveness. Consecutive episodes of nonhypoxic SE in patients ≥14 years old were included. Etiology was classified into acute, remote, progressive, defined electroclinical syndromes, and unknown. Four subcategories of acute etiologies were then considered based on the recent proposal: (1) triggering factors in patients with pre‐existing epilepsy; (2) acute insults to central nervous system (CNS; “acute‐primary CNS”); (3) CNS pathology secondary to metabolic disturbances, systemic infection, or fever (“acute‐secondary CNS”); and (4) drug/alcohol intoxication or withdrawal (“acute‐toxic”). Multinomial logistic regression models were fitted using a backward stepwise selection. Age, sex, consciousness before treatment, SE semiology, SE etiology, SE recurrence, level of disability, and year of presentation were independent variables. The etiological categories of acute‐primary CNS (*p* < .001), acute‐secondary CNS (*p* = .002), progressive (*p* = .010), and unknown (*p* = .018) SE were associated with increased odds of refractory SE. Acute‐primary CNS (*p* < .001), progressive (*p* = .009), and unknown (*p* = .048) SE etiologies were associated with increased odds of superrefractory SE, whereas no association was found with acute‐secondary CNS etiology (*p* = .948). Patients with SE showed different responsiveness to medications according to the underlying cause. Heterogeneity exists within the spectrum of acute symptomatic causes; distinct etiological subcategories may carry different risks of refractoriness and superrefractoriness.

**Key points**

- Patients with SE showed different responsiveness to medications according to the underlying etiology.
- SE due to acute‐primary CNS, acute‐secondary CNS, progressive, and unknown causes was more likely to be refractory.
- Acute‐primary CNS, progressive, and unknown etiologies increased the risk of superrefractoriness.
- There was no association between acute‐secondary CNS etiology and superrefractory SE.

## 1 INTRODUCTION

The rapid cessation of seizure activity is crucial to prevent significant morbidity and mortality associated with status epilepticus (SE), and current treatment protocols suggest an immediate stepwise intervention.^1^ The relationship between the etiology of SE and the response to treatment has been poorly investigated. Besides treatment‐related factors such as timing and appropriateness of the intervention, the cause of SE may also act as a determinant of the refractoriness. In this regard, a more granular classification of acute symptomatic SE into four subcategories (i.e., “acute‐triggering factors in epilepsy,” “acute primary central nervous system [CNS] pathology,” “secondary CNS pathology,” and “acute‐toxic”) has been recently suggested by our group and shown to capture distinct clinical outcomes.^2^, ^3^

This study aimed to explore the potential differences in treatment response in a cohort of adults with nonhypoxic SE within the proposed etiological framework.

## 2 MATERIALS AND METHODS

### 2.1 Participants and collected variables

Consecutive episodes of SE occurring in patients ≥14 years old without hypoxic–anoxic brain injury who were admitted at Baggiovara Civil Hospital (Modena, Italy; September 2015–August 2023) were reviewed. Clinical variables considered for this study included the following: age, sex, level of consciousness before treatment (alert/somnolent, stuporous/comatose),^4^ SE semiology (with prominent motor symptoms, nonconvulsive), SE etiology, SE recurrence (first‐ever vs. recurrent SE episode), level of disability (modified Rankin Scale score) at baseline before SE, year of presentation, and treatment response. Etiology was classified into acute, remote, progressive, defined electroclinical syndromes, and unknown according to the International League Against Epilepsy (ILAE) framework.^1^ Acute etiologies were then classified into four subcategories according to the recent proposal.^2^ Patients with SE as manifestation of defined electroclinical syndromes were excluded, as these etiologies are mostly associated with a distinct course compared to other causes of SE.

Responsive SE was defined as SE that stopped after first‐line therapy with benzodiazepines alone or followed by second‐line treatment with one antiseizure medication (ASM) administered intravenously. Refractory SE (RSE) was defined as persisting SE after treatment with first‐line benzodiazepines and one second‐line ASM. Superrefractory SE (SRSE) was defined as SE that continued or recurred despite the use of anesthetics for >24 h.^5^ RSE and SRSE were also pooled as “nonresponsive” SE. Details about data collection and clinical management can be found in the Supplementary Methods.

The study outcome was treatment responsiveness considered either as a dichotomous (responsive, nonresponsive SE) or nominal (responsive SE, RSE, SRSE) variable.

### 2.2 Statistical analysis

Values are presented as median (interquartile range [IQR]) for continuous variables and as the number (percent) of patients for categorical variables. Comparisons were made through Mann–Whitney test, Kruskal‐Wallis test, or chi‐squared test. Binomial and multinomial logistic regression models were fitted using a backward stepwise selection with variable entry set at *p* = .05 and removal at *p* = .10 to explore the relationship between the etiology of SE and study outcome. Age, sex, consciousness before treatment, SE semiology, SE etiology, SE recurrence, level of baseline disability, and year of presentation were selected as independent variables. As sensitivity analysis, least absolute shrinkage and selection operator (LASSO) logistic regression with 10‐fold cross‐validation was applied to both binary and multinomial outcomes using the same set of candidate predictors; variables retained in the parsimonious LASSO models were re‐entered into the corresponding binomial and multinomial logistic regressions. All statistical models were then verified in the subgroup of patients with first‐ever SE. Results were reported as odds ratio or relative risk ratio with associated 95% confidence intervals. Data analysis was performed using Stata/IC 13.1 (StataCorp). The study is reported according to the STROBE (Strengthening the Reporting of Observational Studies in Epidemiology) guidelines.

### 2.3 Standard protocol approvals and patient consents

The local ethics committee approved the study (556/2018/OSS/AOUMO–RF‐2016‐02361365). All participants or their legal representatives gave informed written consent according to the Declaration of Helsinki.

## 3 RESULTS

A total of 662 episodes of nonhypoxic SE were identified. After the exclusion of 13 episodes of SE in defined electroclinical syndromes, 649 episodes of SE occurring in 579 subjects were included in the analysis. The median age at SE onset was 73 (IQR = 61–81) years, and 275 (42.4%) episodes occurred in males; a plot of participants by age is provided in Figure S1. The prevalent SE etiology was acute symptomatic (60.1%), and within this category, primary and secondary CNS insults were the most common causes. The distribution of etiologies of SE across age strata of participants and vice versa is shown in Figures S2 and S3. In the study cohort, the median Status Epilepticus Severity Score was 3,^2^, ^3^, ^4^ and the median Epidemiology‐Based Mortality Score in Status Epilepticus was 54 (IQR = 28–83). Demographic and clinical characteristics of SE episodes are summarized in Table S1. SE was responsive in 439 (67.6%) cases. Nonresponsive SE included 128 (19.7%) episodes of RSE and 82 (12.6%) episodes of SRSE. Severe impairment of consciousness at onset was more frequent in nonresponsive SE, either RSE or SRSE, than responsive SE. Most episodes of responsive SE were due to symptomatic remote causes (23.5%), whereas acute‐primary CNS pathologies (49.0%) were the most common etiology of nonresponsive SE. The most common causes of RSE were acute‐primary (42.2%) and acute‐secondary (25.8%) CNS insults; the most common causes of SRSE were acute‐primary CNS (59.8%) and progressive (17.1%) pathologies. Demographic and clinical characteristics of SE episodes according to treatment response are summarized in Table S2 (responsive, nonresponsive SE) and Table 1 (responsive SE, RSE, SRSE).

**TABLE 1:** Characteristics of SE episodes by response to treatment: Responsive, refractory, and superrefractory.

Characteristic	Responsive, *n* = 439	Refractory, *n* = 128	Superrefractory, *n* = 82	*p*
Age, years [IQR]	73 [60–81]	71 [59–81]	75 [67–80]	*p* = .628
Sex, *n* (%)				*p* = .296
Female	260 (59.2)	73 (57.0)	41 (50.0)	
Male	179 (40.8)	55 (43.0)	41 (50.0)	
Semiology, *n* (%)				*p* = .169
SE with prominent motor symptoms	227 (51.7)	59 (46.1)	34 (41.5)	
Nonconvulsive SE	212 (48.3)	69 (53.9)	58 (58.5)	
Consciousness, *n* (%)				*p* < .001
Alert or somnolent/confused	357 (81.3)	54 (42.2)	18 (21.9)	
Stuporous or comatose	82 (18.7)	74 (57.8)	64 (78.1)	
Etiology, *n* (%)				*p* < .001
Acute				
Acute‐TFE	47 (10.7)	5 (3.9)	1 (1.2)	
Acute‐primary CNS	81 (18.4)	54 (42.2)	49 (59.8)	
Acute‐secondary CNS	95 (21.6)	33 (25.8)	7 (8.5)	
Acute‐toxic	15 (3.4)	2 (1.6)	1 (1.2)	
Remote	103 (23.5)	9 (7.0)	7 (8.5)	
Progressive	85 (19.4)	20 (15.6)	14 (17.1)	
Unknown	13 (3.0)	5 (3.9)	3 (3.7)	
Recurrent SE, *n* (%)	46 (10.5)	15 (11.7)	9 (11.0)	*p* = .922
mRS before SE [IQR]	2 [0–4]	2 [0–4]	1 [0–3]	*p* = .473

*Note*: Data are median [IQR] for continuous variables and *n* (%) for categorical variables. Probability values are unadjusted for multiple comparisons.

Abbreviations: CNS, central nervous system; IQR, interquartile range; mRS, modified Rankin scale; SE, status epilepticus; TFE, triggering factors in epilepsy.

Sankey diagrams display the clustering of treatment response by SE etiology according to either ILAE classification and the proposed subcategories in responsive and nonresponsive SE (Figure S4) and in responsive SE, RSE, and SRSE (Figure 1).

**FIGURE 1:**
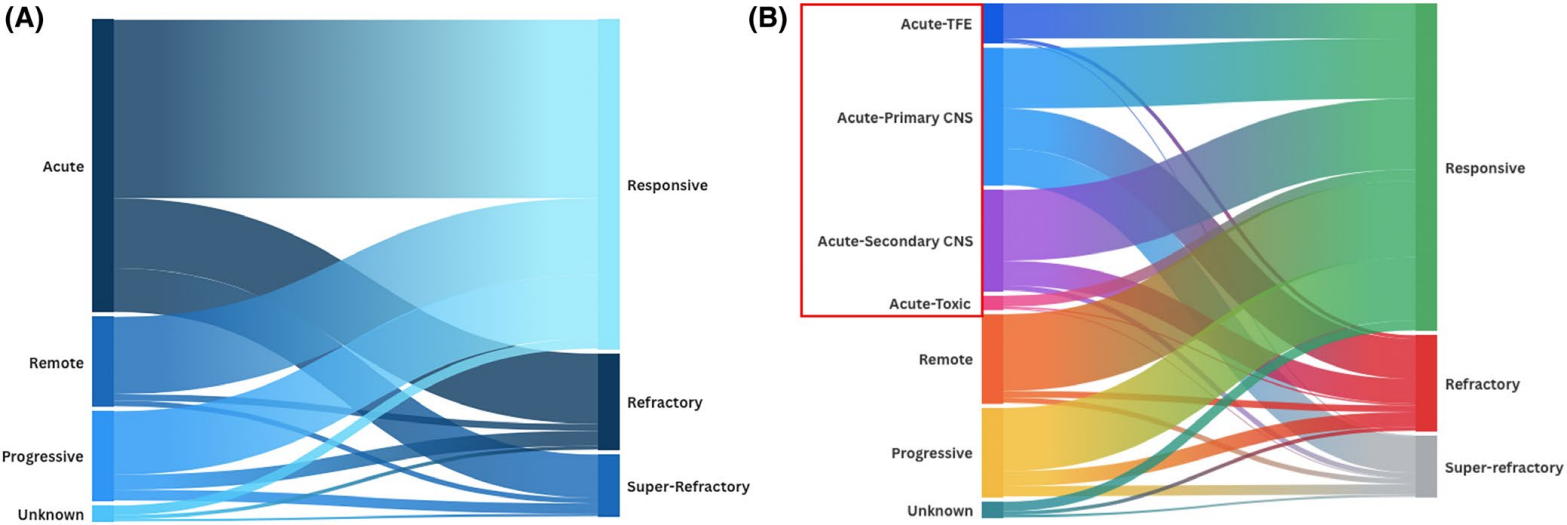
Etiologies of status epilepticus (SE) and response to treatment clustering of treatment response by etiology of SE. In panel B, acute etiologies are grouped within the red rectangle and classified into four subcategories: (1) withdrawal, or low levels, or inappropriate prescription of antiseizure medications, febrile illnesses, or sleep deprivation in patients with pre‐existing epilepsy (“Acute‐TFE”); (2) acute insults to central nervous system (CNS; “Acute‐Primary CNS”); (3) CNS pathology secondary to metabolic disturbances, systemic infection, or fever (“Acute‐Secondary CNS”); and (4) drug/alcohol intoxication or withdrawal (“Acute‐Toxic”).^2^ TFE, triggering factors in epilepsy.

The logistic regression analysis showed that groups of acute‐primary CNS, acute‐secondary CNS, progressive, and unknown SE etiology were significantly associated with nonresponsive SE (Table S3). In the multinomial logistic regression model, acute‐primary CNS, acute‐secondary CNS, progressive, and unknown SE etiology were significantly associated with increased odds of RSE; acute‐primary CNS, progressive, and unknown SE etiology were significantly associated with increased odds of SRSE (Table S4), whereas no association was found with acute‐secondary CNS etiology. In LASSO sensitivity analyses, SE etiology and level of consciousness before treatment were retained as relevant predictors. Logistic and multinomial models refitted using these variables yielded effect estimates that closely matched those of the main analyses (Tables S5 and S6). Results of the analyses performed including only patients with first‐ever SE were consistent with the results obtained in the full cohort (Tables S7–S10).

## 4 DISCUSSION

In a cohort of patients ≥14 years old with nonhypoxic SE, acute primary and secondary CNS pathologies carried the highest risk of treatment refractoriness. Conversely, patients with symptomatic SE due to withdrawal of or low levels of ASMs or other triggering factors in epilepsy, acute toxic causes, or remote CNS insults had the lowest likelihood of being refractory. Among the subgroups of acute symptomatic etiologies, primary CNS pathologies were the only to be independently associated with SRSE. Similarly, SE due to progressive and unknown causes resulted in high risk of being refractory and superrefractory. LASSO sensitivity models and subgroup analyses in first‐ever SE episodes confirmed the robustness of the associations.

Besides confirming low medication levels in epilepsy patients and remote symptomatic etiologies as conditions usually highly responsive to benzodiazepines and subsequent intravenous ASMs,^6^ the study built upon the current, limited and unsettled, body of knowledge about the relationship between acute etiology and treatment response. The findings underline the vast heterogeneity of acute symptomatic causes of SE as drivers of refractoriness and contribute to understanding the inconsistency in available evidence. Whereas acute symptomatic etiology reached statistical significance as the most common cause of refractory compared to nonrefractory SE in some studies,^7^, ^8^, ^9^ it was not associated with treatment refractoriness in others.^10^, ^11^ Such discrepancy is likely because this category encompasses a wide spectrum of causes, including relatively benign conditions such as ASM withdrawal in people with epilepsy, and others that are less responsive to treatment such as structural lesions of the brain. The findings also reinforce from a new perspective the opportunity of refining the current classification of SE etiology. A granular framework encompassing distinct subgroups has been already found to better appreciate differences in the risk of in‐hospital mortality and poor outcome at discharge and to more accurately stratify the probability of post‐SE epilepsy compared to the “umbrella” category of acute etiology.^2^, ^3^ This “split” categorization of acute SE causes has been recently adopted and integrated into the ACARD score, a prognostic model of 30‐day mortality after SE.^12^

The current study was designed simply to identify associations and generate hypotheses, and it was not able to infer causation and investigate potential underlying mechanisms. Although the likelihood of SE cessation has been shown to decrease with time, not all cases of prolonged SE develop resistance to treatment at the same rate. Other factors could contribute to poor treatment response, and etiology may be among these. Robust preclinical and clinical data suggest that the development of resistance to benzodiazepines can be etiologically determined. In experimental studies, diazepam was successful in terminating kainate‐induced SE, whereas it failed to terminate SE in pilocarpine‐induced SE models, and this was likely attributable to the reduction in the surface expression of γ2 subunit‐containing γ‐aminobutyric acid type A (GABA_A_) receptors and the synaptic inhibition mediated by GABA_A_ receptors.^13^ In a clinical setting, the presence of acute brain injury was independently associated with a poor response to benzodiazepines, even in cases of their prompt administration, whereas patients with chronic structural lesions responded faster.^14^ Although very limited evidence exists outside benzodiazepines,^15^ it might be speculated that etiology can also contribute to the risk of poor response to drugs following the recommended first‐line treatment. Within the framework of the burden model of SE, primary and secondary CNS insults and progressive brain conditions might be accompanied by higher structural damage and more severe impairment of brain functional reserve, which in turn can increase the extent of decompensation, alter the excitatory/inhibitory balance, and negatively affect the response to the antiseizure effects of the medications.^16^ In this regard, the robust association between acute primary CNS insult and either RSE or SRSE and the link of acute secondary CNS insult with RSE but not SRSE suggest a gradient within acute damage to the brain, with lesional conditions likely to be more impactful or harmful and to have more detrimental or sustained effects on cerebral functioning than metabolic ones.

Despite being very preliminary and purely exploratory, these findings could open new lines of research about SE management. Some SE etiologies are less responsive than others, and some are extremely difficult to treat, but so far, they are treated all the same. In this regard, a “Stage 1 Plus” has been recently suggested as a stage that includes SE due to primary CNS disorders and requires a synergistic approach from the start, with a combination of first‐ and second‐line treatment.^17^ The concept of a Stage 1 Plus points to the importance of identifying the underlying etiology and selecting correct and sufficiently aggressive treatment early to avoid ending in RSE/SRSE. Studies are required to investigate whether tailoring timing and duration of treatment with either ASMs or anesthetics according to the cause of SE may reduce the duration of abnormally prolonged seizure activity and whether etiology‐dependent protocols may improve clinical outcomes.

This study provided novel insights into the relationship between etiology and treatment refractoriness of SE and identified evidence‐based differences across subcategories of acute symptomatic causes. The study included a hospital‐based cohort of patients not limited to the intensive care unit, the sample size was large, and statistical models were adjusted for a variety of potential confounding variables, including SE recurrence and year of SE presentation. Different shortcomings need, however, to be acknowledged. The recruitment at a single tertiary care center may hamper the generalizability of the results, and the low number of SE episodes due to acute intoxication and unknown causes could not allow drawing definitive conclusions about these categories. The data collection performed in a real‐world setting may have resulted in potential sources of bias, although a prospective registry is maintained at the center and clinical variables are prospectively assessed and recorded using a dedicated form. Age did not independently affect the distribution of responsive SE, RSE, and SRSE, but the population was quite elderly and the low number of patients in the younger age groups may have masked an age dependency. Although the relationship between age at SE onset and etiology was not specifically investigated, being outside the scope of the current study, both variables were included in the multivariate models, and the distribution of the etiological subcategories of SE across different age strata was mapped. It is noteworthy that the presence of prominent motor symptoms has been shown to lead to a faster recognition of SE as opposed to nonconvulsive SE,^14^ and it cannot be excluded that the association of refractoriness and etiology was only the result of delay in recognition and treatment. In this regard, although the time from SE onset to treatment was not considered in the statistical analyses, as it was not available throughout the entire cohort, the meaningful differences observed after the adjustment for SE semiology played against this hypothesis. An intrinsic heterogeneity necessarily exists within each group of etiologies, and additional efforts should be oriented to stratify the degree of severity of any individual causes of SE. Future studies including more variables and data are warranted to validate the findings in independent cohorts.

## 5 CONCLUSIONS

The underlying biologic background represents a major prognostic contributor in SE and is linked with the responsiveness to medications.^18^ So far, most of our understanding of the mechanisms of treatment refractoriness comes from experiments in animals rather than from observations in humans. The clinical heterogeneity of SE represents a potentially rich source of knowledge and understanding about how the many forms of SE start and develop.

## AUTHOR CONTRIBUTIONS

Simona Lattanzi planned and designed the study, performed the statistical analyses, interpreted the data, and drafted and revised the manuscript. Giada Giovannini and Niccolò Orlandi acquired and interpreted the data and revised the manuscript. Francesco Brigo and Eugen Trinka revised the manuscript. Stefano Meletti planned and designed the study, interpreted the data, contributed to the inaugural draft, and revised the manuscript. All authors approved the final submitted version.

## FUNDING INFORMATION

None.

## CONFLICT OF INTEREST STATEMENT

S.L. has received speaker's or consultancy fees from Angelini Pharma, Eisai, GW Pharmaceuticals, Hikma Pharmaceuticals, Medscape, NewBridge Pharmaceuticals, and UCB Pharma and has served on advisory boards for Angelini Pharma, Arvelle Therapeutics, Bial, Eisai, GW Pharmaceuticals, Hikma Pharmaceuticals, Rapport Therapeutics, and UCB Pharma outside the submitted work. E.T. has received personal honoraria for lectures and educational activities from EVER Pharma, Marinus, Arvelle, Angelini Pharma, Alexion, Argenx, Medtronic, Biocodex, Bial‐Portela & Ca, NewBridge, GL Pharma, GlaxoSmithKline, Boehringer Ingelheim, LivaNova, Eisai, Epilog, UCB Pharma, Biogen, Sanofi, Stoke Therapeutics, Jazz Pharmaceuticals, and Rapport Therapeutics outside the submitted work; his institution has received research grants from Biogen, UCB Pharma, Eisai, Red Bull, Merck, Bayer, the European Union, FWF Österreichischer Fond zur Wissenschaftsforderung Bundesministerium für Wissenschaft und Forschung, and Jubiläumsfond der Österreichischen Nationalbank outside the submitted work. E.T. is cofounder and CMO of PrevEp, Codirector of the European Consortium for Epilepsy Trials, and an Executive Committee member of the Epilepsy Study Consortium. S.M. has received speaker's or consultancy fees from Angelini Pharma, Eisai, GW Pharmaceuticals, Jazz Pharmaceuticals, Eisai, and UCB Pharma and has served on advisory boards for Angelini Pharma, Eisai, UCB Pharma, and Neuraxpharm outside the submitted work. The remaining authors have no conflicts of interest. We confirm that we have read the Journal's position on issues involved in ethical publication and affirm that this report is consistent with those guidelines.

## Supporting information


Table S1.
Table S2.
Table S3.
Table S4.
Table S5.
Table S6.
Table S7.
Table S8.
Table S9.
Table S10.
Figure S1.
Figure S2.
Figure S3.
Figure S4.

## Data Availability

Anonymized data will be shared by request of any qualified investigator.
