# Supplementary material for: Status epilepticus in adults: From etiology to treatment response
Source: Epilepsia. 2026 Aug 13;67(10):e202–8. doi: 10.1002/epi.70446 (PMC13675759; doi:10.1002/epi.70446)
Supplement: Supplementary file 1 [file EPI-67-e202-s001.pdf]

Supplementary Appendix

p2: Supplementary Methods

p4: Table S1. Characteristics of status epilepticus episodes

p5: Table S2. Characteristics of status epilepticus episodes by response to treatment: responsive and non-responsive

p6: Table S3. Association between characteristics of status epilepticus and treatment response: responsive and non-responsive

p7: Table S4. Association between characteristics of status epilepticus and treatment response: responsive, refractory, and super-refractory

p10: Table S5. Logistic regression based on LASSO selected predictors

p11: Table S6. Multinomial regression based on LASSO selected predictors

p8: Table S7. Association between characteristics of status epilepticus and treatment response in first-ever status epilepticus: responsive and non-responsive

p9: Table S8. Association between characteristics of status epilepticus and treatment response in first-ever status epilepticus: responsive, refractory, and super-refractory

p12: Table S9. Logistic regression based on LASSO selected predictors in first-ever status epilepticus

p13: Table S10. Multinomial regression based on LASSO selected predictors in first-ever status epilepticus

p14: Figure S1. Age of participants at onset of status epilepticus

p15: Figure S2. Aetiologies of status epilepticus according to age of participants

p16: Figure S3. Age of participants according to aetiologies of status epilepticus

pp17-18: Figure S4. Aetiologies of status epilepticus and response to treatment

p19: Supplementary References

Supplementary Methods

A prospective registry of cases of status epilepticus (SE) is maintained at the centre, ensuring that clinical variables are prospectively assessed and recorded in a dedicated form by the neurologists or neuro-intensivists taking care of the individuals with SE.

The definition of SE proposed by the ILAE was adopted.[1] Clinical variables considered for this study included: age, sex, level of consciousness before treatment (alert/somnolent, stuporous/comatose),[2] semiology of SE, aetiology of SE, SE recurrence (first-ever versus recurrent SE episode), level of disability at baseline before SE as assessed by the modified Rankin Scale, and response to treatment.

Semiology was classified into SE with prominent motor symptoms and without prominent symptoms, i.e. non-convulsive SE (NCSE);[1] NCSE was diagnosed according to the Salzburg EEG criteria.[3]

Aetiology was classified into acute, remote, progressive, defined electroclinical syndromes, and "unknown" (i.e., cryptogenic) according to the ILAE framework.[1] Acute aetiologies were then classified into four subcategories according to the recent proposal: i) withdrawal, low levels, or inappropriate prescription of antiseizure medications (ASMs), or sleep deprivation in patients with pre-existing epilepsy ("acute-triggering factors in epilepsy"); ii) acute primary CNS pathology, including cerebrovascular diseases, active CNS infections, or head trauma ("acute-primary CNS"); iii) secondary CNS pathology, including metabolic disturbances (e.g., electrolyte imbalances, glucose imbalance, organ failure, acidosis, renal failure, hepatic encephalopathy), systemic infection, or fever ("acute-secondary CNS"); and iv) drug or alcohol intoxication and withdrawal ("acute-toxic").[4] In case of presumed competing aetiologies, classification followed physician's judgment regarding the leading cause. Patients with SE as manifestation of defined electroclinical syndromes were excluded as these aetiologies are mostly associated with a distinct course compared to other causes of SE.

According to response to treatment, SE was classified into responsive, refractory, and super-refractory. Responsive SE was defined as a SE that stopped after first-line therapy with benzodiazepines alone or followed by second-line treatment with one ASM administered intravenously. Refractory SE (RSE) was defined as persisting SE after treatment with first-line benzodiazepines and one second-line ASM. Super-refractory SE (SRSE) was defined as a SE that continued or recurred despite the use of anesthetics for longer than 24 hours.[5] Treatment followed the recommendations of international guidelines.[6-8] First-line therapy involved intravenous benzodiazepines, repeated if needed. For benzodiazepine-refractory SE, second-line treatment with levetiracetam, lacosamide, valproic acid, or phenytoin was initiated. In RSE, nonsedating antiseizure drugs were added and continuous propofol or midazolam infusion was started. Anaesthetics were titrated to termination of seizures or the establishment of burst suppression at the physician's discretion.

To monitor seizure activity, 30-minute spot EEG or on-demand continuous EEG (c-EEG) with at least 21 electrodes was performed from 2015 to 2018; from 2020 onward, cEEG indications expanded to the management of RSE, suspected NCSE, and altered mental status with lack of clinical explanation. For SE with prominent motor symptoms, SE onset and treatment response were defined clinically; for NCSE, SE onset and treatment response were defined by EEG.

Data on Withdrawal of Life Sustaining Treatment (WLST) such as the time (during or after SE) and reason were reviewed; the definition of RSE in this cohort was not affected by WLST in any case as no patients were undertreated as far as first- and second-line treatments based on WLST.

Table S1. Characteristics of status epilepticus episodes

	Status epilepticus episodes (n=649)
**Age, years**	73 [61-81]
**Sex, n (%)**	
Female	374 (57.6)
Male	275 (42.4)
**Semiology, n (%)**	
Status epilepticus with prominent motor symptoms	320 (49.3)
Non-convulsive status epilepticus	329 (50.7)
**Consciousness, n (%)**	
Alert or somnolent/confused	429 (66.1)
Stuporous or comatose	220 (33.9)
**Aetiology, n (%)**	
Acute	
Acute-TFE	53 (8.2)
Acute-primary CNS	184 (28.4)
Acute-secondary CNS	135 (20.8)
Acute-toxic	18 (2.8)
Remote	119 (18.3)
Progressive	119 (18.3)
Unknown	21 (3.2)
**Recurrent status epilepticus**	70 (10.8)
**mRS score before status epilepticus**	2 [0-4]

Data are median [IQR] for continuous variables and n (%) for categorical variables.

Abbreviations: CNS=central nervous system, IQR=interquartile range, mRS=modified Rankin scale, TFE=triggering factors in epilepsy.

Table S2. Characteristics of status epilepticus episodes by response to treatment: responsive and non-responsive

	Responsive (n=439)	Non-responsive (n=210)	p value
**Age, years**	73 [60-81]	74 [62-81]	p=0.896
**Sex, n (%)**			p=0.233
Female	260 (59.2)	114 (54.3)	
Male	179 (40.8)	96 (45.7)	
**Semiology, n (%)**			p=0.077
Status epilepticus with prominent motor symptoms	227 (51.7)	93 (44.3)	
Non-convulsive status epilepticus	212 (48.3)	117 (55.7)	
**Consciousness, n (%)**			p<0.001
Alert or somnolent/confused	357 (81.3)	72 (34.3)	
Stuporous or comatose	82 (18.7)	138 (65.7)	
**Aetiology, n (%)**			p<0.001
Acute			
Acute-TFE	47 (10.7)	6 (2.9)	
Acute-primary CNS	81 (18.4)	103 (49.0)	
Acute-secondary CNS	95 (21.6)	40 (19.1)	
Acute-toxic	15 (3.4)	3 (1.4)	
Remote	103 (23.5)	16 (7.6)	
Progressive	85 (19.4)	34 (16.2)	
Unknown	13 (3.0)	8 (3.8)	
**Recurrent status epilepticus**	46 (10.5)	24 (11.4)	0.715
**mRS before status epilepticus**	2 [0-4]	2 [0-4]	p=0.957

Data are median [IQR] for continuous variables and n (%) for categorical variables.

p values are unadjusted for multiple comparisons. Abbreviations: CNS=central nervous system, IQR=interquartile range, mRS=modified Rankin scale, TFE=triggering factors in epilepsy.

Table S3. Association between characteristics of status epilepticus and treatment response: responsive and non-responsive

	Adjusted OR (95% CI)	p value
**[a]Aetiology**		
Acute-primary CNS	5.68 (3.14-10.28)	<0.001
Acute-secondary CNS	2.48 (1.34-4.59)	0.004
Progressive	3.19 (1.68-6.05)	<0.001
Unknown	5.09 (1.73-14.97)	0.003
**[b]Consciousness**		
Stuporous/comatose	6.36 (4.27-9.48)	<0.001
**Recurrent status epilepticus**	1.93 (1.03-3.62)	0.042

Values are from binary logistic regression model fitted using a backward stepwise selection with variable entry set at p=0.05 and removal at p=0.10 (see text for details).

[a]Reference is remote aetiology. [b]Reference is alert/somnolent.

Abbreviations: CNS=central nervous system, CI=confidence interval, OR=odds ratio.

Table S4. Association between characteristics of status epilepticus and treatment response: responsive, refractory, and super-refractory

	Adjusted RRR (95% CI)	p value
**Refractory status epilepticus**		
**[a]Aetiology**		
Acute-primary CNS	4.32 (2.25-8.26)	<0.001
Acute-secondary CNS	2.93 (1.49-5.75)	0.002
Progressive	2.61 (1.26-5.40)	0.010
Unknown	4.21 (1.28-13.89)	0.018
**[b]Consciousness**		
Stuporous/comatose	4.77 (3.04-7.48)	<0.001
**Super-refractory status epilepticus**		
**[a]Aetiology**		
Acute-primary CNS	5.37 (2.38-12.09)	<0.001
Acute-secondary CNS	0.97 (0.33-2.80)	0.948
Progressive	3.45 (1.35-8.78)	0.009
Unknown	4.72 (1.01-22.00)	0.048
**[b]Consciousness**		
Stuporous/comatose	12.13 (6.56-22.41)	<0.001

Values are from multinomial logistic regression model with responsive status epilepticus treated as the base outcome and fitted using a backward stepwise selection with variable entry set at p=0.05 and removal at p=0.10 (see text for details).

[a]Reference is remote aetiology. [b]Reference is alert/somnolent.

Abbreviations: CNS=central nervous system, CI=confidence interval, RRR=relative-risk ratio.

Table S5. Logistic regression based on LASSO selected predictors

	Adjusted OR (95% CI)	p value
**[a]Aetiology**		
Acute-TFE	0.91 (0.32-2.59)	0.854
Acute-primary CNS	4.79 (2.51-9.14)	<0.001
Acute-secondary CNS	2.25 (1.13-4.49)	0.021
Acute-toxic	1.43 (0.34-5.97)	0.625
Progressive	2.89 (1.43-5.86)	0.003
Unknown	4.41 (1.46-13.33)	0.009
**[b]Consciousness**		
Stuporous/comatose	6.53 (4.39-9.73)	<0.001

[a]Reference is remote aetiology. [b]Reference is alert/somnolent.

Abbreviations: CNS=central nervous system, CI=confidence interval, OR=odds ratio; TFE=triggering factors in epilepsy.

Table S6. Multinomial regression based on LASSO selected predictors

	Adjusted RRR (95% CI)	p value
**Refractory status epilepticus**		
**[a]Aetiology**		
Acute-TFE	1.29 (0.40-4.19)	0.667
Acute-primary CNS	4.90 (2.22-10.83)	<0.001
Acute-secondary CNS	3.33 (1.48-7.51)	0.004
Acute-toxic	1.65 (0.31-8.73)	0.558
Progressive	2.96 (1.25-7.01)	0.013
Unknown	4.79 (1.33-17.19)	0.016
**[b]Consciousness**		
Stuporous/comatose	4.78 (3.05-7.51)	<0.001
**Super-refractory status epilepticus**		
**[a]Aetiology**		
Acute-TFE	0.35 (0.04-3.07)	0.343
Acute-primary CNS	4.50 (1.82-11.12)	0.001
Acute-secondary CNS	0.81 (0.26-2.52)	0.715
Acute-toxic	1.13 (0.12-10.94)	0.919
Progressive	2.89 (1.04-7.98)	0.041
Unknown	3.96 (0.81-19.39)	0.090
**[b]Consciousness**		
Stuporous/comatose	12.08 (6.53-22.33)	<0.001

[a]Reference is remote aetiology. [b]Reference is alert/somnolent.

Abbreviations: CNS=central nervous system, CI=confidence interval, RRR=relative-risk ratio, TFE=triggering factors in epilepsy.

Table S7. Association between characteristics of status epilepticus and treatment response in first-ever status epilepticus: responsive and non-responsive

	Adjusted OR (95% CI)	p value
**[a]Aetiology**		
Acute-primary CNS	6.77 (3.47-13.23)	<0.001
Acute-secondary CNS	2.86 (1.38-5.93)	0.005
Progressive	3.75 (1.80-7.84)	<0.001
Unknown	5.85 (1.86-18.42)	0.003
**[b]Consciousness**		
Stuporous/comatose	6.67 (4.32-10.28)	<0.001
**[c]Sex**	1.51 (0.99-2.29)	0.055

Values are from binary logistic regression model fitted using a backward stepwise selection with variable entry set at p=0.05 and removal at p=0.10 (see text for details).

[a]Reference is remote aetiology. [b]Reference is alert/somnolent. [c]Reference is female.

Abbreviations: CNS=central nervous system, CI=confidence interval, OR=odds ratio.

Table S8. Association between characteristics of status epilepticus and treatment response in first-ever status epilepticus: responsive, refractory, and super-refractory

	Adjusted RRR (95% CI)	p value
**Refractory status epilepticus**		
**[a]Aetiology**		
Acute-primary CNS	6.25 (2.83-13.80)	<0.001
Acute-secondary CNS	3.80 (1.66-8.72)	0.002
Progressive	3.42 (1.43-8.21)	0.006
Unknown	6.28 (1.75-22.47)	0.005
**[b]Consciousness**		
Stuporous/comatose	4.91 (3.02-8.01)	<0.001
**[c]Sex**	1.36 (0.86-2.17)	0.192
**Super-refractory status epilepticus**		
**[a]Aetiology**		
Acute-primary CNS	7.63 (2.75-21.23)	<0.001
Acute-secondary CNS	1.22 (0.34-4.36)	0.764
Progressive	4.55 (1.46-14.26)	0.009
Unknown	4.97 (0.79-31.42)	0.088
**[b]Consciousness**		
Stuporous/comatose	12.28 (6.32-23.87)	<0.001
**[c]Sex**	1.86 (1.04-3.34)	0.036

Values are from multinomial logistic regression model with responsive status epilepticus treated as the base outcome and fitted using a backward stepwise selection with variable entry set at p=0.05 and removal at p=0.10 (see text for details).
[a]Reference is remote aetiology. [b]Reference is alert/somnolent. [c]Reference is female.
Abbreviations: CNS=central nervous system, CI=confidence interval, RRR=relative-risk ratio.

Table S9. Logistic regression based on LASSO selected predictors in first-ever status epilepticus

	Adjusted OR (95% CI)	p value
**[a]Aetiology**		
Acute-TFE	1.14 (0.27-4.79)	0.858
Acute-primary CNS	7.99 (3.49-18.27)	<0.001
Acute-secondary CNS	3.37 (1.40-8.10)	0.007
Acute-toxic	2.44 (0.52-11.49)	0.258
Progressive	4.42 (1.82-10.73)	0.001
Unknown	6.90 (1.98-24.04)	0.002
**[b]Consciousness**		
Stuporous/comatose	6.66 (4.31-10.28)	<0.001
**[c]Sex**	1.51 (0.99-2.30)	0.054

[a]Reference is remote aetiology. [b]Reference is alert/somnolent. [c]Reference is female.

Abbreviations: CNS=central nervous system, CI=confidence interval, OR=odds ratio; TFE=triggering factors in epilepsy.

Table S10. Multinomial regression based on LASSO selected predictors in first-ever status epilepticus

	Adjusted RRR (95% CI)	p value
**Refractory status epilepticus**		
**[a]Aetiology**		
Acute-TFE	2.12 (0.44-10.22)	0.351
Acute-primary CNS	9.30 (3.13-27.60)	<0.001
Acute-secondary CNS	5.66 (1.85-17.30)	0.002
Acute-toxic	3.23 (0.51-20.39)	0.212
Progressive	5.09 (1.61-16.12)	0.006
Unknown	9.36 (2.13-41.10)	0.003
**[b]Consciousness**		
Stuporous/comatose	4.93 (3.02-8.05)	<0.001
**[c]Sex**	1.37 (0.86-2.19)	0.184
**Super-refractory status epilepticus**		
**[a]Aetiology**		
Acute-TFE	Not reliably estimable	0.987
Acute-primary CNS	6.50 (2.09-20.17)	0.001
Acute-secondary CNS	1.04 (0.26-4.06)	0.961
Acute-toxic	1.58 (0.14-17.54)	0.709
Progressive	3.86 (1.11-13.36)	0.033
Unknown	4.21 (0.63-28.36)	0.139
**[b]Consciousness**		
Stuporous/comatose	12.11 (6.23-23.54)	<0.001
**[c]Sex**	1.87 (1.04-3.35)	0.036

[a]Reference is remote aetiology. [b]Reference is alert/somnolent.

Abbreviations: CNS=central nervous system, CI=confidence interval, OR=odds ratio; TFE=triggering factors in epilepsy. [c]Reference is female.

Figure S1. Age of participants at onset of status epilepticus

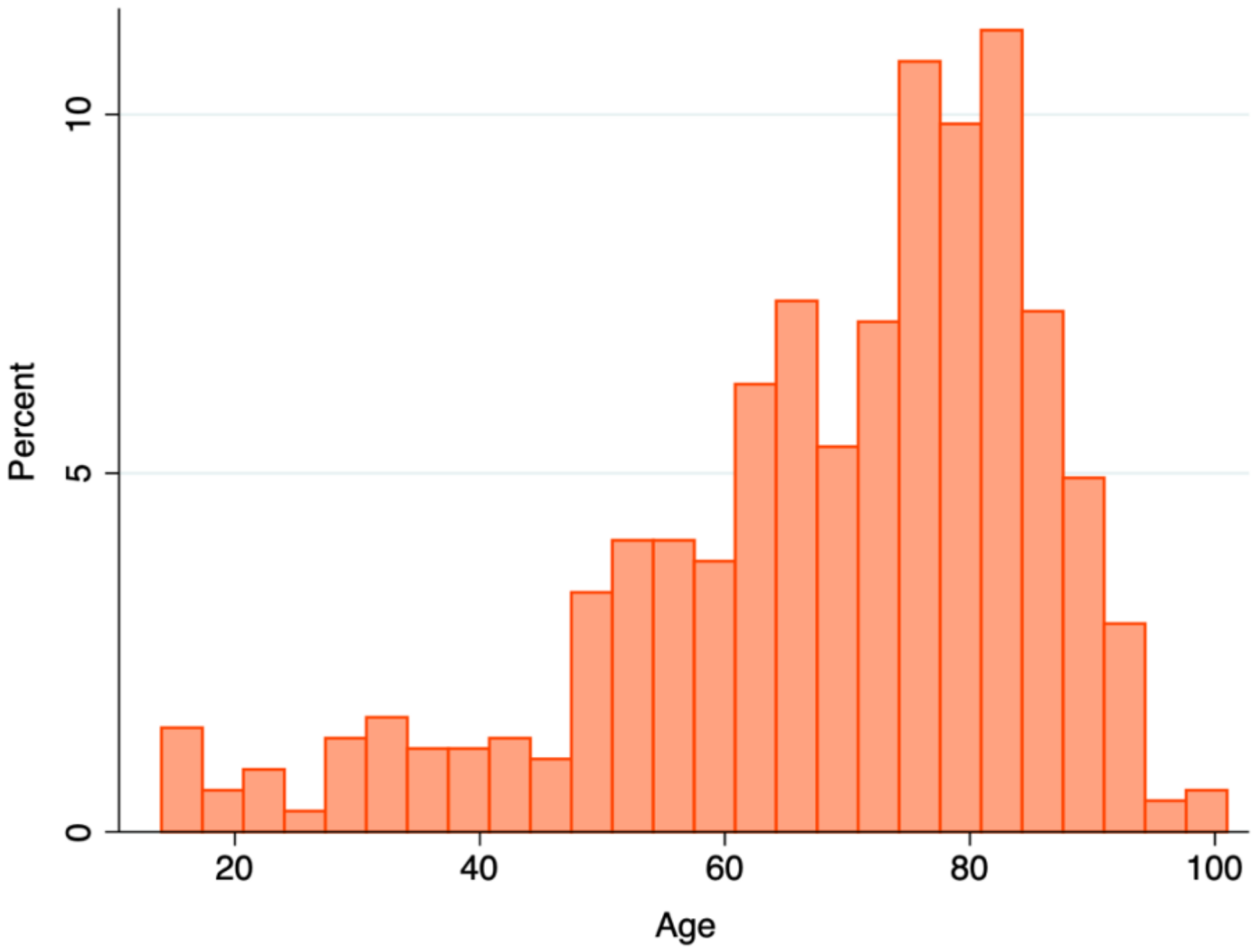

Distribution of age at onset of status epilepticus across the study cohort. Median age at status epilepticus onset: 73 [interquartile range 61-81] years.

Figure S2. Aetiologies of status epilepticus according to age of participants

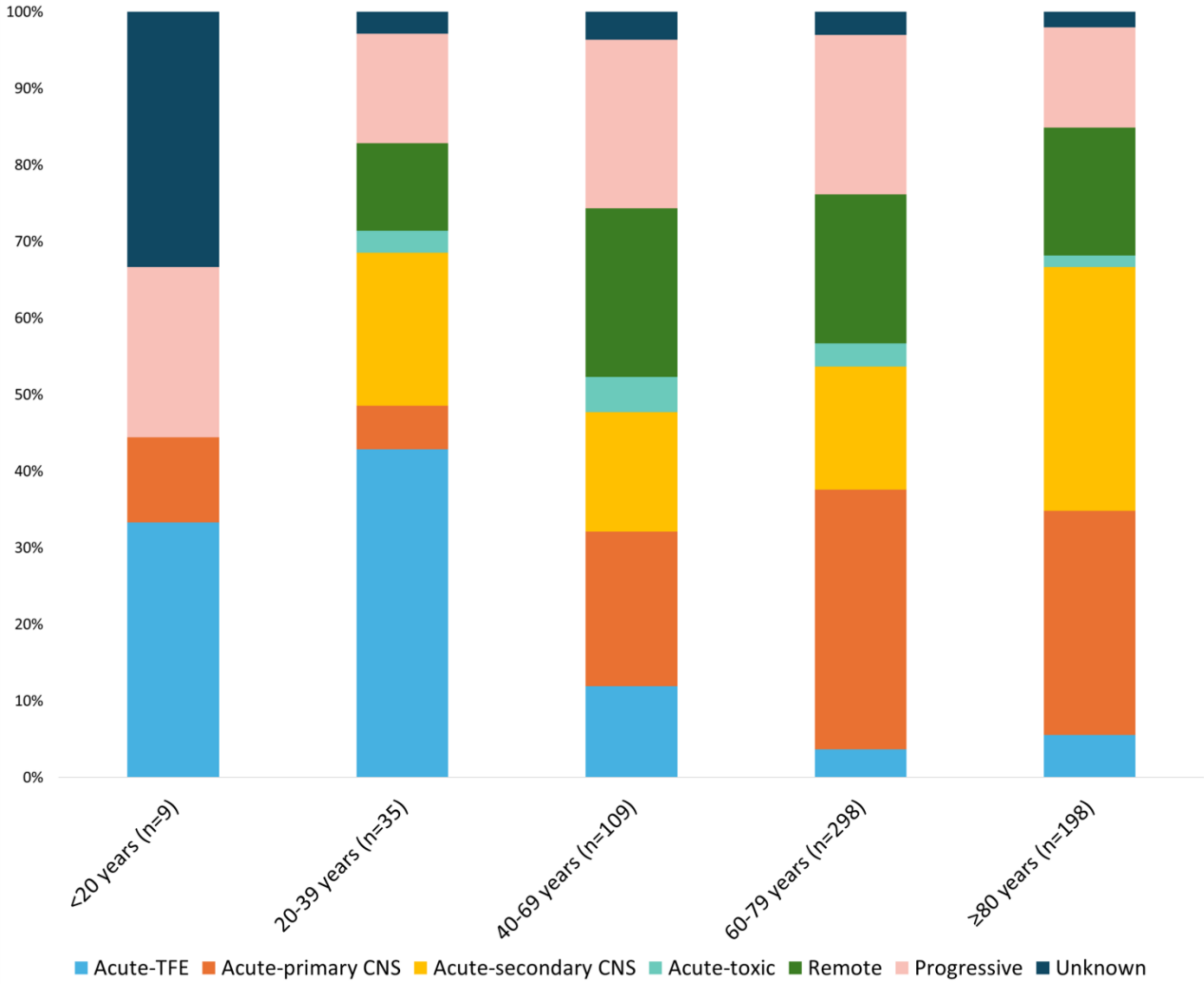

Proportions of participants by the aetiological categories of status epilepticus within age strata.

Figure S3. Age of participants according to aetiologies of status epilepticus

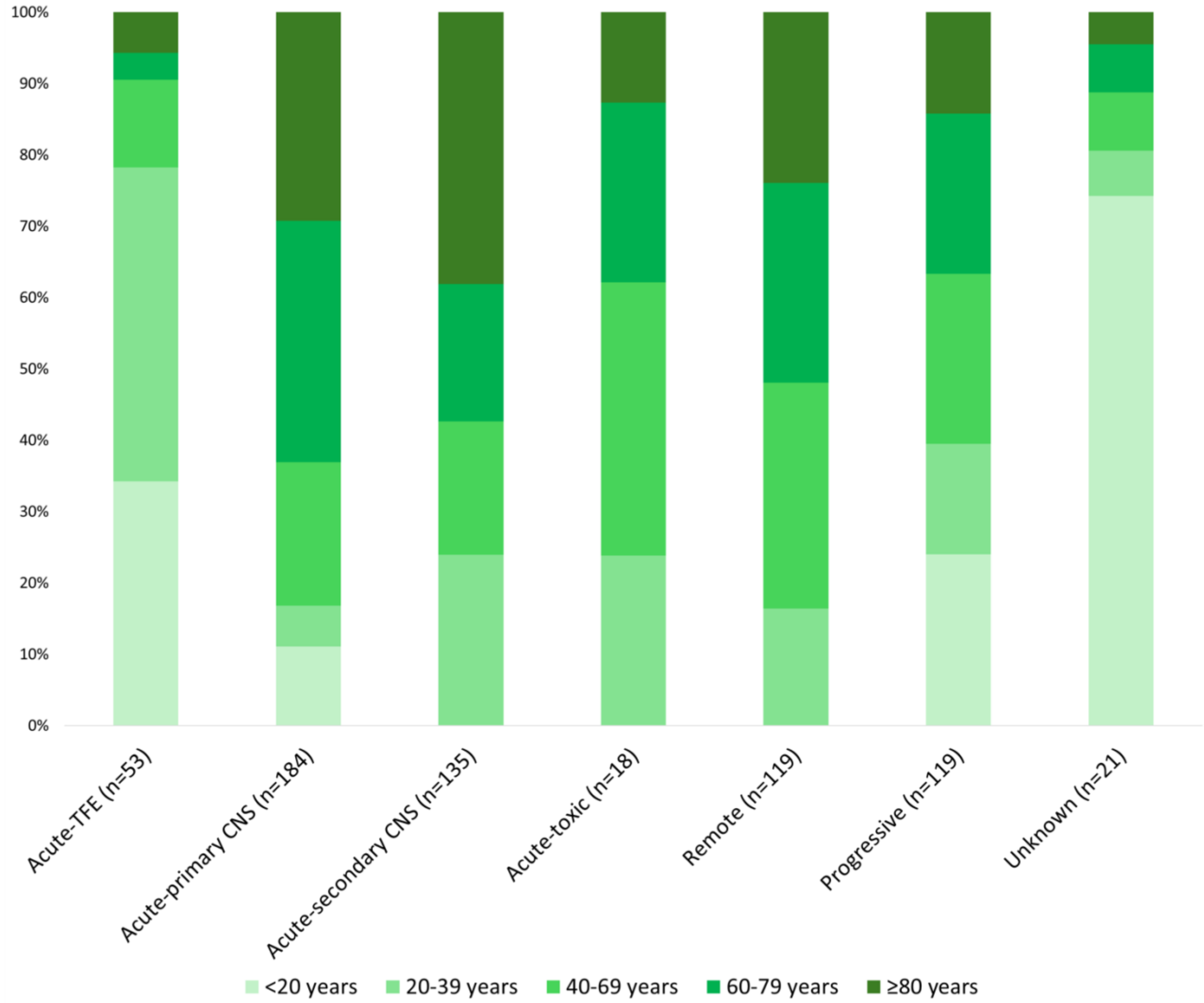

Proportions of participants by age strata within the aetiological categories of status epilepticus.

Figure S4. Aetiologies of status epilepticus and response to treatment

A.

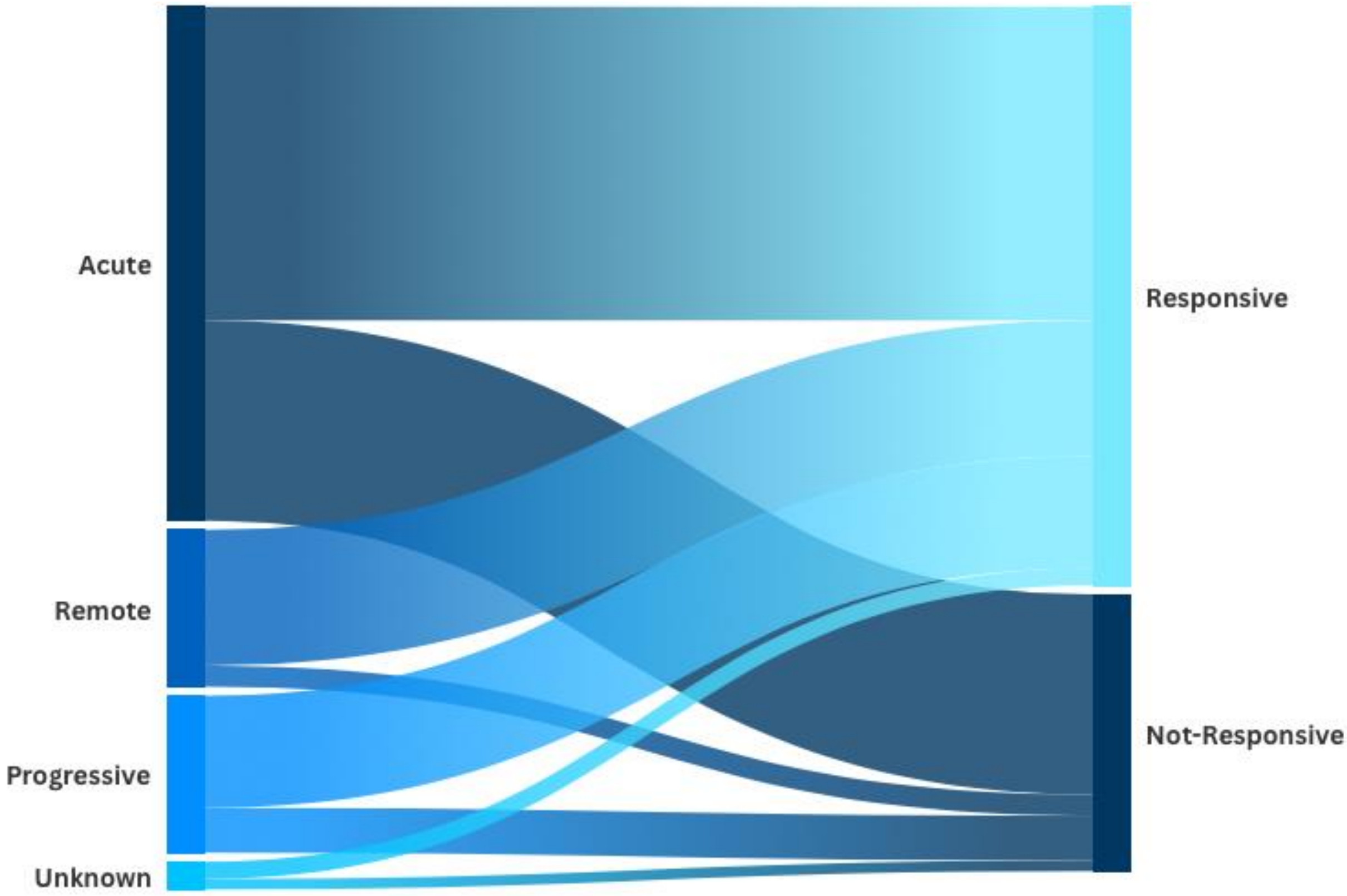

B.

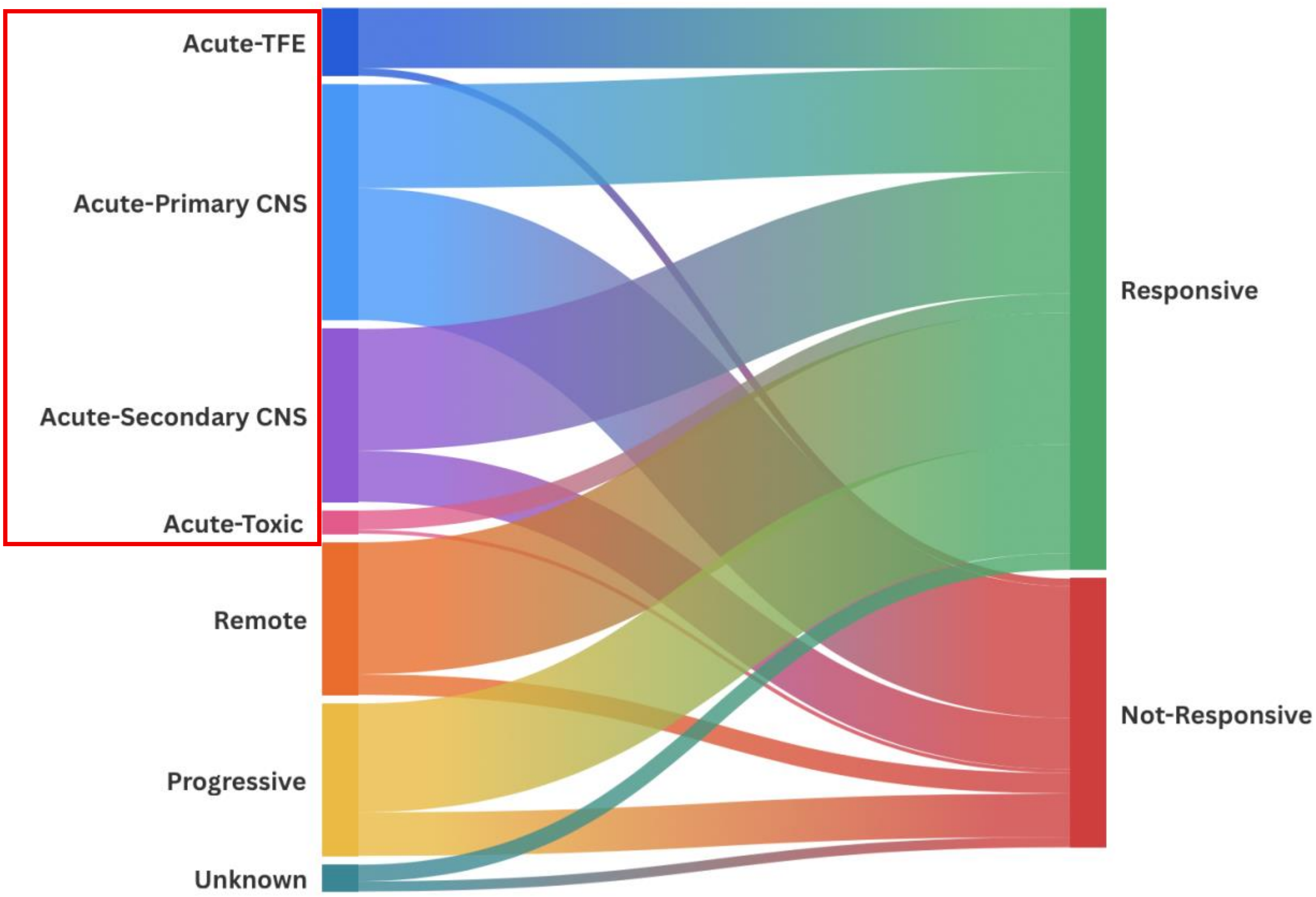

Clustering of treatment response by aetiology of SE. Non-responsive SE includes refractory and super-refractory SE. In panel B, acute aetiologies are grouped within the red rectangle and classified into four subcategories: i) withdrawal, or low levels, or inappropriate prescription of antiseizure medications, febrile illnesses, or sleep deprivation in patients with pre-existing epilepsy ("acute-TFE"); ii) acute insults to central nervous system (CNS) ("acute-primary CNS"); iii) CNS pathology secondary to metabolic disturbances, systemic infection, or fever ("acute-secondary CNS"); and iv) drug/alcohol intoxication or withdrawal ("acute-toxic").[4]

Abbreviations: CNS=central nervous system, SE=status epilepticus, TFE=triggering factors in epilepsy.

Supplementary References

1. Trinka E, Cock H, Hesdorffer D, Rossetti AO, Scheffer IE, Shinnar S, et al. A definition and classification of status epilepticus—report of the ILAE Task Force on Classification of Status Epilepticus. Epilepsia. 2015;56:1515-23.
2. Rossetti AO, Logroscino G, Milligan TA, Michaelides C, Ruffieux C, Bromfield EB. Status Epilepticus Severity Score (STESS): a tool to orient early treatment strategy. J Neurol 2008;255:1561-6.
3. Leitinger M, Beniczky S, Rohracher A, Gardella E, Kalss G, Qerama E, et al. Salzburg consensus criteria for non-convulsive status epilepticus–approach to clinical application. Epilepsy Behav. 2015;49:158-63.
4. Lattanzi S, Giovannini G, Brigo F, Orlandi N, Trinka E, Meletti S. Acute symptomatic status epilepticus: Splitting or lumping? A proposal of classification based on real-world data. Epilepsia. 2023;64:e200-6.
5. Lowenstein DH, Bleck T, Macdonald RL. It's time to revise the definition of status epilepticus. Epilepsia. 1999;40:120-2.
6. Brophy GM, Bell R, Claassen J, Alldredge B, Bleck TP, Glauser T, et al. Guidelines for the evaluation and management of status epilepticus. Neurocrit Care 2012;17:3-23.
7. Glauser T, Shinnar S, Gloss D, Alldredge B, Arya R, Bainbridge J, et al. Evidence-Based Guideline: Treatment of Convulsive Status Epilepticus in Children and Adults: Report of the Guideline Committee of the American Epilepsy Society. Epilepsy Curr. 2016;16:48-61.
8. Minicucci F, Ferlisi M, Brigo F, Mecarelli O, Meletti S, Aguglia U, et al. Management of status epilepticus in adults. Position paper of the Italian League against Epilepsy. Epilepsy Behav. 2020;102:106675.
